# Evaluation of the Influence of a Hydrogel Containing AMPD on the Stability of Tetracycline Hydrochloride

**DOI:** 10.3390/pharmaceutics13091381

**Published:** 2021-09-01

**Authors:** Agnieszka Kostrzębska, Adrianna Złocińska, Witold Musiał

**Affiliations:** 1 Department of Physical Chemistry and Biophysics, Faculty of Pharmacy, Wroclaw Medical University, ul. Borowska 211A, 50-556 Wrocław, Poland; agnieszka.kostrzebska@umed.wroc.pl; 2 Laboratory of Elemental Analysis and Structural Research, Faculty of Pharmacy, Wroclaw Medical University, ul. Borowska 211A, 50-556 Wrocław, Poland; adrianna.zlocinska@umed.wroc.pl

**Keywords:** tetracycline hydrochloride, stability, carbomer hydrogel, AMPD

## Abstract

Tetracyclines, as beneficial antimicrobial factors in both local and systemic therapy, are characterized by high instability. The aim of the study was the development of the influence of hydrogel formulation on the tetracycline hydrochloride (TC) level under varying storage conditions. The HPLC, XPRD as well as SEM and macroscopic observations were involved in the study. The TC concentration decreased within ca. two months from 9.37 µg/mL to 4.41 µg/mL in the case of the photoprotected TC solution stored at 23 °C, whereas the decrease in storage temperature did not improve the final level of TC. In the presence of AMPD, the TC level in aqueous solution decreased drastically to ca. 1 µg/mL. Application of a polyacrylic acid derivative enabled conservation of the TC level through the ca. two months. Thus, the use of alcoholamine in the preparation of the TC hydrogel may result in the development of a therapeutic product with a dual action against acne, including antimicrobial activity and saponification of free fatty acids deposited in the follicles.

## 1. Introduction

Tetracyclines are a group of antibiotics with a broad spectrum of antibacterial activity, acting against Gram-negative and Gram-positive bacteria, as well as rickettsiae, chlamydia and some protozoa [1]. In sub-antibacterial doses, they are also used in the treatment of various inflammations, dermatoses, oral cavity diseases, autoimmune diseases such as rheumatoid arthritis or some cancers [2,3]. For many years, both in the oral and topical form, they have been used successfully in the treatment of acne vulgaris caused by strains of *Cutibacterium acnes* [4,5,6].

At the same time, these antibiotics are extremely sensitive to unfavourable external conditions. Factors such as humidity, temperature, light or extreme pH values quickly lead to the degradation of the parent compound. The degradation depending on the conditions leads to the formation of very different products such as 4-epitetracycline (4-ETC), anhydrotetracycline (ATC), 4-epianhydrotetracycline (4-EATC), lumitetracycline (LTC), izotetracycline (ITC) and others [7,8,9,10,11,12]. The decomposition products do not show any antibacterial activity; however, they may be toxic, especially nephrotoxic and phototoxic [8,10,13,14,15,16].

In our study, the stability of TC contained in the hydrogel formulation was determined and compared with the stability of the TC in an aqueous solution. The prepared hydrogels were characterized by slightly acidic pH values in order to ensure greater stability of TC and inhibit the degradation of the antibiotic. All samples prepared were subjected to various external conditions, such as changing temperature and photoprotection or lack thereof.

HPLC analysis was used to determine changes in TC concentration in the prepared solutions and hydrogels. Furthermore, SEM microscopic analysis of the freeze-dried hydrogel samples was performed to assess the distribution of TC in the hydrogels. The optical assessment of changes taking place in the tested preparations, such as colour change, the appearance of sediments or turbidity, was also carried out.

Hydrogel preparations were prepared using a polyacrylic acid—carbomer (Carbopol 980 NF) —which was neutralized with 2-amino-2-methyl-1,3-propandiol (AMPD). The former studies on the interaction of alcoholamines with model skin sebum included the evaluation of the influence of the alcoholamines on the saponification of the sebum free fatty acids of model sebum prepared according to the available literature [17,18,19]. The model skin sebum used in these studies was recognized by Stefaniak and Harvey as an example of a preparation that corresponds fairly well to the composition of the natural sebum of human skin [20]. The resulting amine soap, by binding water, loosens the mass of sebum and may facilitate their removal from the pilosebaceous apparatus. It has also been shown that combining alcoholamines with anionic polymers extends their effect on model skin sebum and allows the development of a product with a pH appropriate for the skin and providing greater stability to the active substances contained in the hydrogel [21]. The present study goes further, to reveal the possibility of the application of the selected alcoholamine—AMPD—in the presence of tetracycline hydrochloride. The combined activity of antibiotic and AMPD may be beneficial in terms of local eradication of *C. acnes*; however, the stability of the TC may be hindered by AMPD; therefore, we have implemented the TC stability study in the presence of AMPD in combined preparations containing both of the components. Hair follicles can be exploited by the transfollicular route of drug delivery, but as Meidan et al. have noted, the natural sebum remaining in them is a barrier that can hinder this transport [22]. Alcoholamine, proposed in our study, as a substance that can support cleansing the hair follicles from sebum residual in them, can be used as a factor improving the local action of antibacterial preparations used in acne, as well as supporting transfollicular transport. Moreover, appropriate selection of the type of carbomer and alcoholamine, as well as their mutual proportions, enables the development of gel preparations with various parameters, such as viscosity or pH values of the system, adjusted to the various active substances used.

The subject of our considerations was to evaluate the concentration changes of tetracycline hydrochloride, implemented into the hydrogel containing anionic polymer—polyacrylic acid, doped with AMPD. The evaluation of the tetracycline stability in the hydrogel is a crucial factor for the development of a medicinal product with a dual action—cleansing the skin and hair follicles from the layers of skin sebum residual in them, while at the same time having an antibacterial effect.

## 2. Materials and Methods

### 2.1. Reagents

Carbopol 980 NF, polyacrylic acid crosslinked with allyl pentaerythritol (Lubrizol, Wickliffe, OH, USA), AMPD (Sigma Aldrich, Poznan, Poland), TC (Sigma Aldrich) and demineralized, bi-distilled water were used to prepare the hydrogel. Formic acid (Sigma Aldrich), acetonitrile (Sigma Aldrich) and demineralized, bi-distilled water were used in the chromatographic analysis.

### 2.2. Preparation of Hydrogels and Solutions

The hydrogel H and three solutions—A1, A2 and A3 were prepared.

Two batches of the hydrogel H with identical composition and weight of 100 g were prepared containing Carbopol 980 NF, AMPD and demineralized water. After 24 h conditioned at 5 °C in a fridge, 0.2 g TC were added to each hydrogel (Table 1).

The content of Carbopol 980 NF and AMPD was selected so that the pH value of the resulting hydrogel was close to neutral. For a hydrogel with 1% of these components, the pH value was 6.652; SD = 0.029. The pH value was measured five times using a pH meter CPC-505 Elmetron (accuracy up to ±0.002 pH, Elmetron Sp.j., Zabrze, Poland) and pH electrode IJ44At HT (Elmetron Sp.j., Zabrze, Poland).

After the addition of TC, each hydrogel H was mixed for 16 min in order to obtain proper homogenization of the product in an Alpina MR500 automatic pharmacy mixer (Alpina Polska Sp. z o.o., Konin, Poland) at the lowest speed 1 to avoid air entrainment. Both batches of the hydrogel H were divided into a three equal parts—H1, H2 and H3. Parts H1 were stored at 5 °C in a fridge with photoprotection. Parts H2 were stored at 23 °C with photoprotection, and parts H3 were stored at 23 °C and in daylight (Table 1).

Three aqueous solutions containing 0.2 g TC were prepared (Table 1). The pH value of these solutions was 2.998, SD = 0.013. The pH value was measured five times using a pH meter CPC-505 Elmetron and pH electrode IJ44At HT. 

Solution A1 was stored in a fridge at 5 °C with photoprotection. Solution A2 was stored at 23 °C with photoprotection, and solution A3 was stored at 23 °C in daylight.

### 2.3. HPLC Analysis of Hydrogels and Solutions

#### 2.3.1. Samples Preparation

A total of 0.5 g of each parts of hydrogels—H1, H2 and H3—was dissolved in 99.5 g of water. The samples were mixed for 20 min on a magnetic stirrer at 900 rpm, until the gel was completely dissolved in the water. For this purpose, Arex Digital Pro, a heating magnetic stirrer (Velp Scientifica, Usmate (MB), Italy), was used. The samples were not heated. From all the obtained solutions, 1 mL was taken four times and analysed by HPLC giving a total of 8 measurements for several parts: H1, H2 and H3. 

Similarly, 0.5 g of the solutions A1–A3 were weighted and diluted in 99.5 g water. From each obtained solution, 1 mL was taken five times and was analysed by HPLC.

Chromatographic observations were carried out for 63 days at various time intervals.

#### 2.3.2. Chromatographic System

HPLC analysis was carried out on a Thermo Scientific Dionex UltiMate 3000 (Dionex Corporation, Sunnyvale, CA, USA) equipped with a pump LPG-3400SD, column oven TCC-3000SD, detector DAD-3000 and autosampler WPS-3000TSL. Chromatographic separations were carried out using column RP-18 LiChroCART, 125 mm × 3 mm, 5 µm (Merck, Darmstadt, Germany) at a temperature of 40 °C. The mobile phase consisted of 0.1% formic acid in water (A) and 0.1% formic acid in acetonitrile (B). The flow rate was 1.0 mL/min with the following gradient elution: starting at 7% mobile phase B and holding for 0.5 min, reaching 50% in 4 min and 95% in 4.5 min and holding for 1 min. From 5.5 min, the gradient returned to 7% mobile phase B and stopped after 7 min in this concentration. The injection volume of the sample was 10 µL, and detection was performed at 280 nm wavelength. The commercial TC of various concentrations was used to prepare the calibration curve (y = 0.2625x − 0.0771, R^2^ = 0.998).

### 2.4. Macroscopic Observations of Changes in the Solutions and Hydrogel Samples

Macroscopic observations were carried out for 63 days at the same time intervals and on the same samples as the chromatographic observations. Colour changes of the hydrogels and solutions as well as appearance of the sediments or turbidity were assessed. The colour change was shown under the same lightning conditions. Observations were made using the Huawei P30 lite camera, 48 megapixel, 27 mm wide angle f/1.8 lens, 1/2.0″ sensor, 0.8 µm pixels, PDAF (Huawei, Shenzhen, China). 

### 2.5. SEM Study of Freeze-Dried Hydrogel Samples

For SEM study, a separate hydrogel sample with the composition in accordance with the hydrogel H was prepared. The sample was divided into three equal parts and was stored for 30 days under the storage conditions given in Table 1. On day 31, samples were frozen and then freeze-dried in a freeze-dryer Alpha 2-4 LD Plus (Martin Christ Gefriertrocknungsanlagen GmbH, Osterode am Harz, Germany) two times for 24 h. The samples prepared in this way were analysed by SEM (BSE) scanning electron microscope Quanta 650 FEG (Thermo Fisher Scientific Inc., Waltham, MA USA). A hydrogel sample of identical composition but without TC was also freeze-dried under the same conditions and analysed by SEM.

### 2.6. Powder X-ray Diffraction Analysis (XRPD)

The freeze-dried samples of the hydrogels and commercial TC were ground in an agate mortar and placed on a Si low background sample holder with Ø 51.5 mm and Ø 20 mm × 0.5 mm sample cavity. Afterwards, samples were subjected to *X*-ray powder diffraction (XRPD) on a D2 PHASER diffractometer (Bruker, AXS, Karlsruhe, Germany) equipped with a Lynxeye detector Cu Kα 1.2 radiation 1.54184 (Å) under constant conditions 30 kV and 10 mA. The data were collected in the Bragg-Brentano (Θ/2Θ) horizontal geometry from 5° to 70° (2Θ) in steps of 0.02° (2Θ) with 0.5 s/step. The divergence slit was 1.0 mm, and the variable rotation was 15 min^−1^. Samples were measured at 295 K. The data were processed using the software Diffrac.Eva 3.2.

## 3. Results

### 3.1. Evaluation of TC Stability Based on HPLC Analysis

During the observation lasting 63 days, the stability of TC in aqueous solutions and in hydrogels stored in various external conditions was compared. Both the hydrogels H and aqueous solutions A contained the same amount of antibiotic—0.2 g/100 g. Knowing that TC stability depends, among others, on an ambient temperature and an exposure to light, the prepared samples were stored in three different environmental conditions: at the temperature reduced to 5 °C and with photoprotection for samples A1 and H1, at 23 °C with photoprotection for samples A2 and H2 and at 23 °C and exposure to daylight for samples A3 and H3 [7,9,10,11,12,23].

For the first 25 days, observations of the stability of the antibiotic were carried out twice a week for prepared samples. Subsequently, samples were prepared on the 42nd and 63th day from the start of observation. 

**Figure 1:**
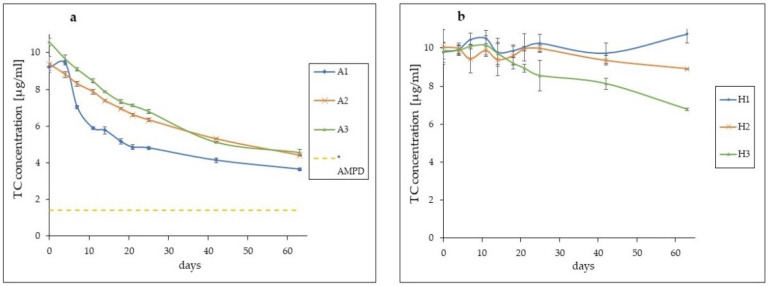
The course of changes of TC concentration. (**a**) changes in aqueous solutions, * the yellow dashed line represents the TC concentration level in the AMPD aqueous solution on 63rd day of observation—1.39 µg/mL; (**b**) changes in the hydrogel samples.

A much more intense decrease in TC concentration was observed in aqueous solutions where the concentration values drop from over 9.2–10.5 µg/mL on the first day of measurements to about 3.6–4.5 µg/mL on day 63 (Figure 1).

In the case of the A1 solution stored in the fridge, a sharp decrease in the value of the TC concentration was observed. On the 7th day of the observation, the TC content of the A1 solution had dropped to 7.03 µg/mL. The disintegration of TC in solutions A2 and A3 during the first 25 days of observation was similar to each other and not as fast as in the case of A1. The TC concentration on the 25th day was around 6.33 µg/mL for solution A2 and 6.79 µg/mL for solution A3. For the A1 solution, this value was 4.80 µg/mL. In the following days, the rate of TC degradation decreased. On day 63, the TC concentration for solution A1 was 3.64 µg/mL, for A2 was 4.4 µg/mL and for A3 4.56 µg/mL. In the case of the hydrogel H samples, such an intense decrease in TC content was not observed throughout the observation period. For sample H1, the concentration remained at 9.7–10.7 µg/mL. The concentration in the H2 sample slightly decreased, ranging in the range of 9–10 µg/mL, while the strongest decrease was recorded in the H3 sample, where the TC concentration slightly decreased, reaching a value of less than 7 µg/mL after 63 days.

**Figure 2:**
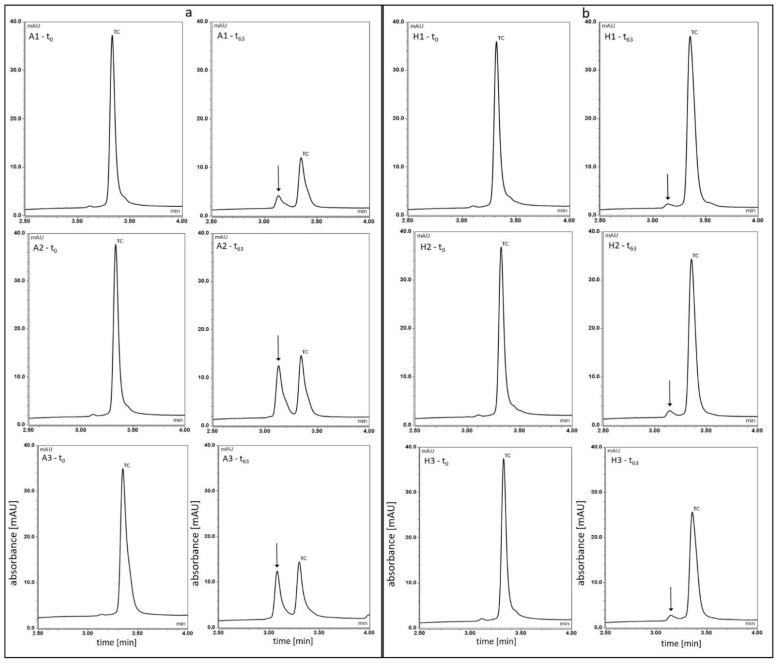
HPLC chromatograms after 0 and 63 days of observation. (**a**) solutions, (**b**) hydrogel samples.

The HPLC chromatograms of all observed samples after 0 and 63 days were also compared (Figure 2).

The HPLC chromatograms of the solutions after 63 days of observation showed a clear reduction in the peak corresponding to TC—retention time 3.3 min, and the appearance of an additional peak, marked with an arrow, preceding the TC peak. At the same time, it was observed that the additional peak was the smallest for solution A1, while for solutions A2 and A3, it was larger and similar. For the hydrogel samples, no such drastic reduction in peak size for TC was observed. The largest reduction in the TC peak occurred in the H3 sample, and the additional peaks for hydrogels H1, H2 and H3 were very small.

### 3.2. Macroscopic Observations of Changes in the Hydrogel Samples and Solutions

Tetracycline is an unstable and easily degraded substance. Depending on the external conditions—temperature, humidity, pH value, exposure to light—different decomposition products are formed and in different concentrations, which is often manifested by a change in colour [9,11,24].

**Figure 3:**
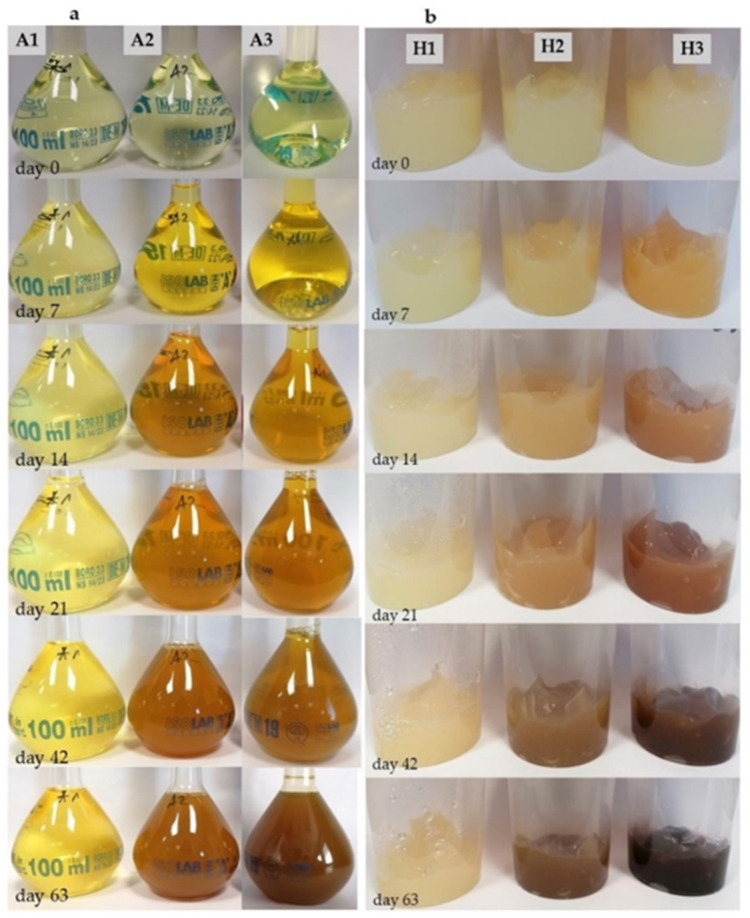
The course of colour changes within 63 days. (**a**) changes in aqueous solutions, (**b**) changes in hydrogel samples.

Starting from the observations on the day 0, all the hydrogel samples and aqueous solutions were the same very light yellow colour (Figure 3). 

Colour changes of the hydrogel samples could be noticed already on the 7th day of the study. The samples stored in 5 °C with photoprotection were unchanged. The samples stored at 23 °C with photoprotection were slightly darkened, while those stored at 23 °C and exposed to daylight darkened the most. This trend in colour changes continued until the end of the observation. On the 63rd day, the samples stored in the low temperature had a slightly stronger yellow colour. The samples stored at 23 °C with photoprotection gradually darkened with an initial orange tinge to a light brown colour by the 63rd day. The most marked change occurred in the samples stored at 23 °C and exposed to the daylight. Over time, the shade of the gel darkened rapidly, going from yellow, orange, light brown to dark brown on the 63rd day.

In case of solutions, colour changes were very similar. All the solutions just after preparation had a light yellow pale shade. On the 7th day of observation, colour changes in the solutions were clearly visible. The least noticeable colour change was observed in the solution A1 kept in the fridge. The solutions A2 and A3, stored at 23 °C, turned an intense yellow colour. Over time, the solution stored in the fridge turned an intense yellow colour, but did not darken until the end of the observation. The solutions A2 and A3 had an orange shade already on the 14th day, which darkened with the passing days, turning into a brown colour. On the 63rd day of the observation, solution A3, exposed to the daylight, was slightly darker.

**Figure 4:**
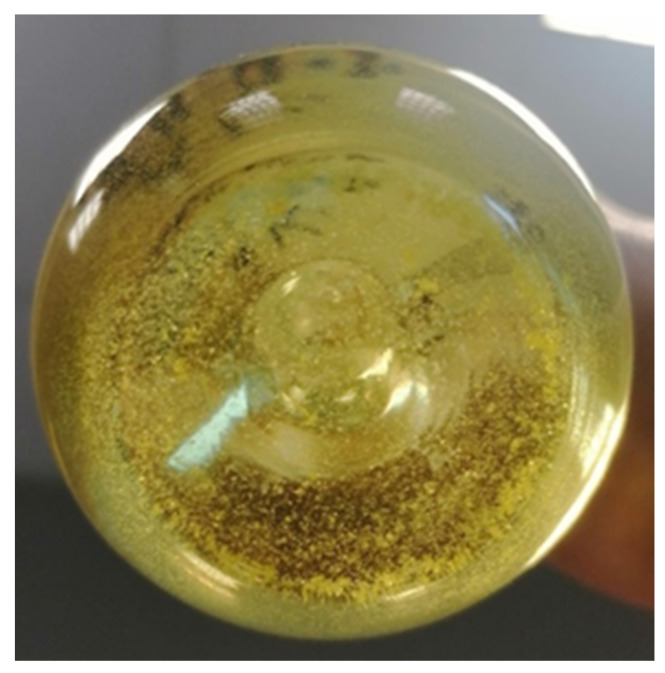
Solution A1—the view of the bottom of the glass vessel with the crystal, yellow sediment on the 14th day of observation.

In addition, after about 2 weeks of observation, turbidity started to appear in solutions A2 and A3, which increased with time. This turbidity did not result in the formation of a crystalline precipitations at the bottom of the glass vessels. In the case of solution A1, turbidity did not occur, but in the first days of observation, the appearance of a yellow, crystalline sediment at the bottom of the vessel was noticed (Figure 4).

### 3.3. Evaluation of SEM Study

**Figure 5:**
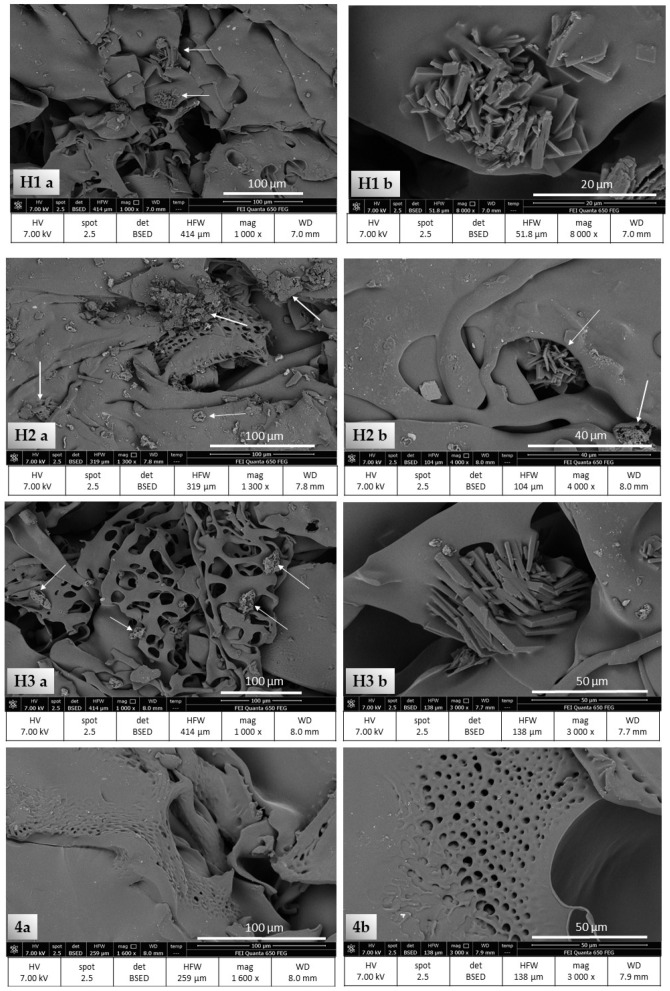
SEM images of the test samples: **1a** and **1b**—sample H1, **2a** and **2b**—sample H2, **3a** and **3b** —sample H3, **4a** and **4b**—hydrogel sample without TC. Images **a**—magnification 1000–1600, **b**—magnification 3000, 4000 and 8000.

The hydrogel samples stored under all three external conditions and the tetracycline free hydrogel sample were freeze-dried and then subjected to SEM analysis (Figure 5).

For the samples containing TC, single amorphous lumps and fine lamellar structures were observed on the hydrogel surface. The images 1a, 2a and 3a show mainly scattered, fine, lumpy structures, as well as small lamellar sets. It seems that the largest of these amorphous structures can be seen in photo 2a for the H2 sample. In the case of the H1 sample, the amorphous structures seem to be smaller, and more fine lamellar structures are noticeable. The images 1b, 2b and 3b show lamellar structures visible at higher magnification, gathered in small conglomerates. All these described structures were not noticed in the images of the hydrogel sample without TC in its composition—4a and 4b.

### 3.4. Powder X-ray Diffraction Analysis (XRPD)

**Figure 6:**
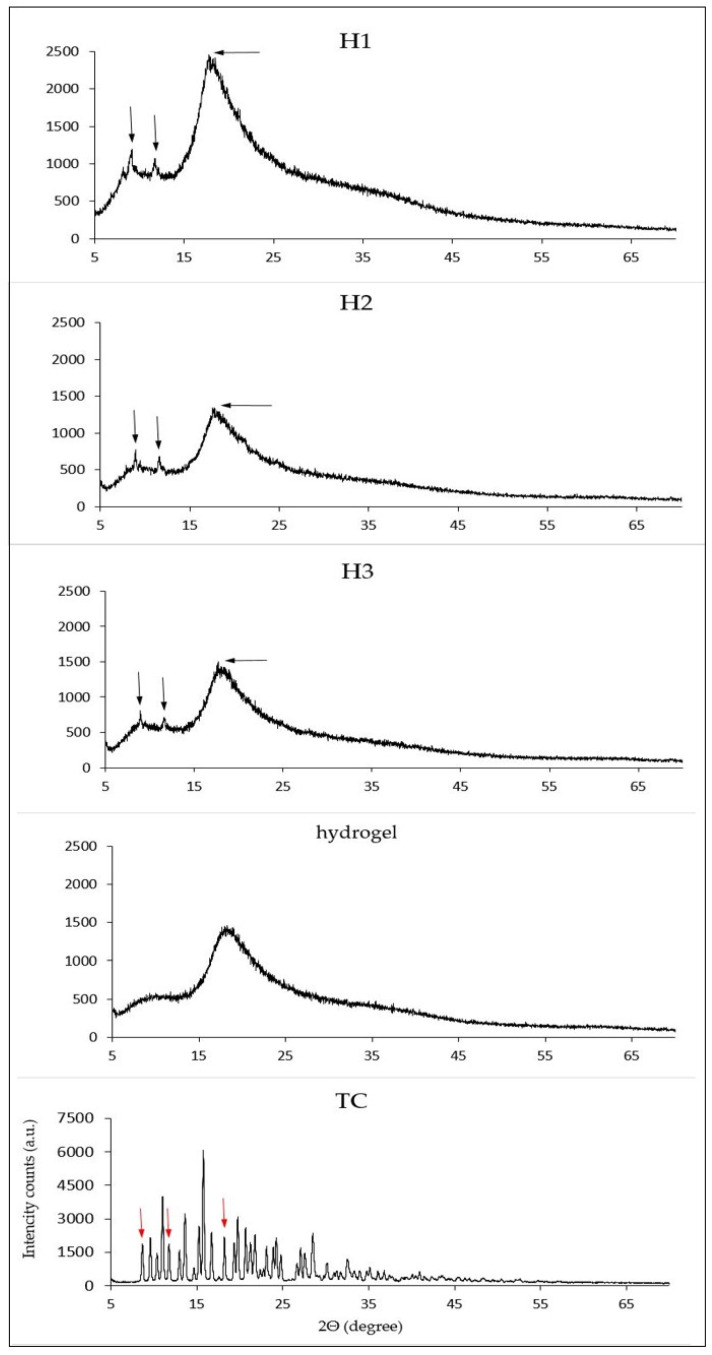
Powder *X*-ray diffraction patterns for investigated samples H1, H2 and H3, hydrogel sample without TC and commercial TC.

The freeze-dried samples of hydrogels containing TC and the sample of hydrogel without TC were subjected to powder *X*-ray diffraction analysis. The commercial TC that was used to prepare the samples was also analysed for comparison. Obtained diffraction patterns are presented in Figure 6.

In the case of diffraction patterns in the samples with hydrogels containing TC, an amorphous halo is visible which is close to the pattern of the hydrogel without TC. They are distinguished from this pattern by the presence of three small, sharp peaks at reflection angles of about 8.9° 2Θ, 11.60° 2Θ and 17.7° 2Θ marked with black arrows. In the diffraction pattern for commercial TC, peaks at similar reflection angles can be noticed; however, they cannot be registered on the diffractogram for a hydrogel without TC (Table 2).

## 4. Discussion

Tetracycline antibiotics are unstable and degrade easily under unfavourable conditions. High humidity and temperature, extreme pH values, the presence of metals and exposure to light accelerate the processes of TC decomposition, and depending on the above conditions, various decomposition products are formed, e.g., 4-ETC, ATC and its toxic epimer, lumitetracycline and other derivatives [7,8,9,10,11,12,23,25,26]. The degradation of the TC molecule very often proceeds with a colour change. Commercial TC powder has a light yellow colour which may change depending on environmental conditions. In an alkaline environment, when exposed to light, TC becomes dark red to maroon, possibly due to the formation of quinones [8,27,28,29]. In a neutral or acidic environment, TC darkens quickly under the influence of temperature, high humidity and light, changing its colour from yellow to orange, grey as a result of degradation products of the compound. The phenomenon of TC decomposition in medical products stored in various external conditions and the appearance of a dark colour were demonstrated in the research by Walton et al. and also other researchers [9,11,24].

This study presents hydrogels that may form the basis for the future development of a dual-action medical preparation. The first of the active ingredients of the preparation is the antibacterial TC; the second is the alcoholamine AMPD, which builds the structure of hydrogels and also actively reacts with the components of the model skin sebum, as shown in previous studies [17]. In such designed hydrogel systems, AMPD can be used as a component that facilitates the cleansing of the pilosebaceous apparatus of the skin from sebum deposits, thus supporting the antibacterial effect of TC against bacteria inhabiting the hair follicle and causing local inflammation. 

The prepared hydrogels were exposed to variable external factors such as light and temperature to evaluate the stability of TC. At the same time, aqueous TC solutions were prepared in order to compare the drug stability in the aqueous environment subjected to the same variable external conditions.

HPLC analysis showed that there was a much greater decrease in TC concentration in the aqueous solutions compared to TC contained in hydrogels. Table 3 shows the percentage of drug residue in the tested samples according to the HPLC analysis.

A significant decrease in TC concentration was observed in all three aqueous solutions. It was the fastest in the A1 solution stored at low temperature and with photoprotection. The decrease in TC content by about half was recorded in this solution on the 18th day of observation, while in the solutions A2 and A3, similar values were noticed on the 42nd day. The reason for such changes may be the precipitation of a crystalline, yellow precipitate only in the A1 solution already in the first days of observation, and the reason for crystallization may be the fact that the solubility of TC in water decreases with a decrease in temperature, which Varanda et al. presented in their research [30]. Additionally, Younis et al. showed that in an aqueous solution of TC, a crystalline precipitate of tetracycline hexahydrate occurs, which is less soluble in water than TC [25]. Moreover, Caira with the team analysed the crystalline precipitate from the hot solution of TC, confirming the structure of tetracycline hexahydrate [31]. Thus, the low temperature, combined with the changes taking place within the tetracycline molecule, could cause the appearance of a crystalline precipitate in the A1 solution. On the 63rd day of observation, all solutions showed a decrease in TC concentration by over 50%, which indicates high instability of the antibiotic in aqueous solutions.

The decrease in TC concentration in the hydrogel samples was much smaller. The greatest stability of the drug was noted in the H1 samples, although its concentration values showed slight fluctuations. Perhaps they were caused by minor changes in drug distribution in the hydrogel by local TC precipitation and formation of small aggregates, as indicated by SEM analysis. Maintaining the proper homogenization of the drug in a hydrophilic gel and preventing this type of phenomena should be the subject of additional research. Despite these small changes, the highest TC stability was demonstrated in the H1 samples. Low storage temperature and limited exposure to light significantly increase the durability of TC in the preparation. A slightly greater decrease in TC content in the preparation was found in the case of H2 samples where, after 63 days of observation, about 88.6% of the active substance remained in the hydrogels. Samples H3 exposed to the light and stored at 23 °C showed by far the worst durability. Here, the TC content dropped to less than 69%.

The stability of TC in the studied samples, apart from the light and temperature, was also influenced by the pH value of the prepared forms. The pH value of solutions A1, A2 and A3 due to dissolved TC oscillated around 2.998. At such low pH values, the leading phenomenon in TC transformations is epimerization, which occurs most quickly at pH values 3–5 or, according to some other researchers, 2–6, while in solutions with a pH value below 2, the main decomposition product is ATC [7,32,33,34]. The pH values of hydrogels were about 6.65, which could significantly slow down the processes of TC molecule decomposition.

The analysis of HPLC spectra, besides the changes in concentration of the parent compound, showed the appearance of a new product in the analysed samples (Figure 2). For all aqueous solutions and hydrogel samples, on the 63rd day of observations, in addition to a significant decrease in the size of the TC peak, the appearance of an additional peak located just before the TC peak was noted. It has been marked with a black arrow in Figure 2. According to the HPLC analyses of tetracyclines carried out by numerous research teams, it can be assumed that this peak corresponds to the product resulting from the epimerization of tetracycline, i.e., 4-epitetracycline [9,24,34,35,36,37]. Both the type of product and its content in the studied samples require confirmation with additional analyses.

In the case of solutions, the peak is much larger than in hydrogels, which indicates a much stronger tendency to transformations and possible epimerization of TC, and greater instability of the parent product in an acidic environment. Among the solutions, the smallest peak appeared in solution A1 which again confirms that the low temperature has an inhibitory effect on the unfavourable TC transformations. The smaller size of this peak in relation to the peaks in a solutions A2 and A3 may also be caused by the formation of a crystalline precipitate in this solution and thus a reduction in the amount of TC remaining in the solution. However, the proportions between the new product peaks and the corresponding TC peaks in individual solutions suggest that the TC transformations in solution A1 occur to a lesser extent. In the case of samples H1, H2 and H3, the peaks of the TC transformation product on the 63rd day are very small, and the smallest one appeared in sample H1. The reported results and observations may indicate the fact that the phenomena of epimerization and degradation of TC occur much slower in hydrogel products, and TC retains greater stability.

The observations of the colour changes of all solutions, carried out simultaneously with the analysis of the concentration changes, showed that until the end of the observation period, the colour of the A1 solution did not change as much as in the case of the other solutions. On the 63rd day, the colour was more intense but still yellow. This fact suggests that low temperature and limited access to light significantly inhibit the course of TC decay. Moreover, the phenomenon of TC precipitation in the form of a crystalline sediment could inhibit the process of antibiotic degradation. 

In the solutions A2 and A3, no crystalline sediment was observed at the bottom, while the solutions became increasingly turbid with time. On the 63rd day of observation, the turbidity level for solution A3 was slightly higher compared to solution A2. It also achieved a darker brown colour. The appearance of a darkening turbidity or sediment may prove the decomposition of TC that progresses under the influence of temperature and exposure to light [24].

In the case of hydrogel samples, similar colour changes were noted after 63 days of observation. The H1 samples remained yellow with only a slightly stronger shade. The H3 samples exposed to the light were dark brown, while those stored in the dark remained light brown. This indicates that an exposure of the preparations to the light and the higher storage temperature for sample other than 1 increases the extent of degradation products’ role in the TC colour change [24]. Liang and his research team suggest that the colour change of tetracycline from yellow to brown is not due to conversion to ATC or 4-EATC. Perhaps responsible for the brown colour of the samples are also other decay products resulting from the action of both light and temperature on the TC molecule [24]. Our observations show that the protection of the antibiotic against direct exposure to light and high temperature significantly inhibits its degradation, both in the case of aqueous solutions and the hydrogel form.

The diffraction patterns of the TC-containing hydrogels obtained during XRPD analysis show an amorphous halo similar to the spectrum of the hydrophilic gel itself, but differing from it by the presence of three small sharp peaks whose reflection angle is similar to those of the commercial TC (Figure 6). Tae Jun Yoon et al. and Junho Chu and his team demonstrated the formation of amorphous TC nanoprecipitates using supercritical anti-solvents [38,39]. The presence of amorphous structures within H1, H2 and H3 hydrogels is confirmed by SEM analysis. However, SEM images of freeze-dried samples of gels containing TC, apart from fine, amorphous lumps, also show the presence of lamellar structures gathered together into small conglomerates. Perhaps within the hydrogels, apart from amorphous structures, the semicrystalline form of TC may also precipitate, which may also be indicated by small sharpened peaks present in the diffraction patterns.

Tetracycline, as a highly unstable antibiotic, creates numerous inactive decomposition products such as 4-ETC or ATC, which, however, are further transformed into 4-epianhydrotetracycline, which is already toxic, leading to reversible kidney damage in Fanconi syndrome and may also cause phototoxicity to the skin [8,10,13,14,15,16]. Numerous studies have shown the presence of 4-EATC in old, out-of-date preparations containing TC, as well as in products stored improperly, which carries a high risk for patients taking them [11,13,14].

Due to the fact that even in technologically pure TC powder, a low presence of its decomposition products is found, and it is practically impossible to obtain pharmaceutical products completely free of them; the acceptable range of its derivatives, such as 4-ETC, ATC and 4-EATC, has been defined [7,11,13,23].

TC is an antibiotic with a broad spectrum of antibacterial activity, thanks to which it is widely used in dermatology, both in oral and topical preparations, as well as in urology, infections of the gastrointestinal tract, respiratory tract and others [1,25,35,40,41]. It also has a non-antibacterial effect and can be used in sub-antimicrobial doses in periodontitis, inflammatory diseases, rheumatoid arthritis, cancers and others [2,3]. Therefore, it is extremely important to develop such pharmaceutical preparations that the stability of TC, and thus its pharmacological activity, are maintained at a stable and high level for the longest possible time.

## 5. Conclusions

The aqueous TC solutions, in both the presence of light and photoprotection stored at 23 °C, presented a similar decomposition of the antibiotic, which reached less than 50% of the original content after 2 months. The aqueous TC system stored at 5 °C with photoprotection showed an even lower level of the antibiotic which can be attributed to the sedimentation of tetracycline hexahydrate. In the presence of AMPD, the TC concentration in the aqueous solution was within 15% of the initial value after 63 days. The application of an anionic polymer in the TC-AMPD system enabled the maintenance of the original TC concentration for ca. 2 months.

The process of degradation and decomposition of TC may be influenced both during the development of pharmaceutical preparation and during its storage. It has been shown that the proper pH of the product, storing it in opaque containers providing protection from light, and protection at low temperatures can significantly extend the shelf life of the antibiotic preparation in the form of a hydrogel. Further steps will determine the activity of AMPD contained in the hydrogel against the components of the model sebum and to elaborate the composition precisely so as to use its cleansing effect fully while maintaining TC stability.

## Figures and Tables

**Table 1:** Composition of samples and storage conditions.

Formulation	Composition				Storage Conditions	
	TC [g]	Water [g]	Carbopol 980 NF [g]	AMPD [g]	Temperature [°C]	Photoprotection
A1	0.20	99.80	-	-	5	Yes
A2	0.20	99.80	-	-	23	Yes
A3	0.20	99.80	-	-	23	No
H1	0.20	97.80	1.00	1.00	5	Yes
H2	0.20	97.80	1.00	1.00	23	Yes
H3	0.20	97.80	1.00	1.00	23	No
AMPD sol	0.20	98.80	-	1.00	5	Yes

**Table 2:** The intensities for three visible diffraction peaks for the samples 1, 2, 3 and commercial TC.

Sample	Peak 1 2Θ (°)	I _1_ (a.u)	Peak 2 2Θ (°)	I _2_ (a.u)	Peak 3 2Θ (°)	I _3_ (a.u)
H1	9.15	1193	11.66	1077	17.79	2459
H2	8.91	770	11.60	680	17.73	1346
H3	8.89	803	11.60	707	17.67	1501
TC	8.65	1875	11.74	1886	18.20	2191

**Table 3:** Percent residual TC in solutions and hydrogels based on HPLC analysis, * *n* = 5, ** *n* = 8.

Day	A1% *		A2% *		A3% *		H1% **		H2% **		H3% **	
0	100.00	SD = 0.19	100.00	SD = 0.43	100.00	SD = 0.19	100.00	SD = 0.52	100.00	SD = 0.90	100.00	SD = 0.43
4	102.42	SD = 0.10	93.97	SD = 0.16	91.25	SD = 0.21	101.51	SD = 0.25	99.21	SD = 0.28	100.17	SD = 0.27
7	76.32	SD = 0.08	88.64	SD = 0.12	86.00	SD = 0.09	106.88	SD = 0.35	93.80	SD = 0.72	102.32	SD = 0.13
11	63.94	SD = 0.08	83.88	SD = 0.14	79.78	SD = 0.11	107.67	SD = 0.40	98.31	SD = 0.33	102.96	SD = 0.51
14	62.64	SD = 0.20	78.81	SD = 0.06	74.38	SD = 0.04	99.98	SD = 0.74	93.39	SD = 0.84	98.74	SD = 0.57
18	56.02	SD = 0.15	74.14	SD = 0.06	69.27	SD = 0.12	100.82	SD = 0.33	95.38	SD = 0.49	93.48	SD = 0.29
21	52.60	SD = 0.13	70.60	SD = 0.08	67.15	SD = 0.08	102.72	SD = 0.73	99.12	SD = 0.13	90.75	SD = 0.20
25	52.16	SD = 0.08	67.57	SD = 0.09	64.17	SD = 0.10	104.89	SD = 0.48	99.35	SD = 0.18	86.80	SD = 0.80
42	44.85	SD = 0.12	56.53	SD = 0.05	48.33	SD = 0.04	99.47	SD = 0.55	93.00	SD = 0.25	82.49	SD = 0.30
63	39.51	SD = 0.07	47.01	SD = 0.03	43.08	SD = 0.16	110.02	SD = 0.47	88.60	SD = 0.03	68.79	SD = 0.07

## Data Availability

Not applicable.
